# The Dimensionless Squared Jerk: An Objective Parameter That Improves Assessment of Hand Motion Analysis during Simulated Shoulder Arthroscopy

**DOI:** 10.1155/2018/7816160

**Published:** 2018-07-11

**Authors:** Erica Kholinne, Maulik J. Gandhi, Arnold Adikrishna, Hanpyo Hong, Haewon Kim, Jaesung Hong, In-Ho Jeon

**Affiliations:** ^1^Department of Orthopedic Surgery, St. Carolus Hospital, Jakarta, Indonesia; ^2^Department of Orthopedic Surgery, Asan Medical Center, College of Medicine, University of Ulsan, Seoul, Republic of Korea; ^3^Upper Limb Department, Robert Jones & Agnes Hunt Hospital, Oswestry, England, UK; ^4^Department of Robotics Engineering, Daegu Gyeongbuk Institute of Science and Technology, Daegu, Republic of Korea

## Abstract

**Purpose:**

Attempts to quantify hand movements of surgeons during arthroscopic surgery faced limited progress beyond motion analysis of hands and/or instruments. Surrogate markers such as procedure time have been used. The dimensionless squared jerk (DSJ) is a measure of deliberate hand movements. This study tests the ability of DSJ to differentiate novice and expert surgeons (construct validity) whilst performing simulated arthroscopic shoulder surgical tasks.

**Methods:**

Six residents (novice group) and six consultants (expert group) participated in this study. Participants performed three validated tasks sequentially under the same experimental setup (one performance). Each participant had ten performances assessed. Hand movements were recorded with optical tracking system. The DSJ, time taken, total path length, multiple measures of acceleration, and number of movements were recorded.

**Results:**

There were significant differences between novices and experts when assessed using time, number of movements with average and minimal acceleration threshold, and DSJ. No significant differences were observed in maximum acceleration, total path length, and number of movements with 10m/s^2^ acceleration threshold.

**Conclusion:**

DSJ is an objective parameter that can differentiate novice and expert surgeons' simulated arthroscopic performances. We propose DSJ as an adjunct to more conventional parameters for arthroscopic surgery skills assessment.

## 1. Introduction

There is currently no accepted definition of arthroscopic skills competency or proficiency [1]. This makes it difficult for training institutions to set skills assessments for competency-based training [2, 3]. Broadly these assessments can be categorized as being subjective, objective, or assumption of competence by numbers.

Subjective assessment is the simplest and earliest form of assessment. It follows similar principles to an apprenticeship, where a trainer will give their trainee or apprentice a global assessment [4]. It has been shown that this form of assessment does not reflect the actual level of skill the trainee may possess [2, 5].

To improve the assessment, more objectively based assessment tools have been developed [1, 6–11], whilst remaining feasible and practical [12, 13]. Objective assessment tools described can be broadly defined into quantifiable outcome measurement (such as mean time to perform the task, force measurements, and motion analysis [12, 14–20] or procedural checklists/global ratings scores (GRS) [7, 21–25] (categorical subjective assessment of defined intraprocedural steps).

When a new skill is being learned, a learning curve can be plotted and maintained at a plateau if a skill is continuously practiced [26, 27]. An individual plateau point does not define competence, but it does assume that most novices should achieve the same skills performance plateau of experienced surgeons with continued practice [26, 27].

In shoulder arthroscopy skills evaluation, outcome measures that were able to discriminate skill level on simulators include time to completion of tasks, distance and path traveled by probe, and number of probe collisions [28–31].

Number of movements is difficult to define. It can be described as the number of deliberate movements above a threshold acceleration value. One study considered an arbitrary value of 10 m/s^2^ as the threshold value to detect deliberate hand movements of the surgeon. [12] Another study considered the minimum acceleration value from each participant as the threshold value to detect the deliberate hand movement [16]. Hence, the lack of clarity on the optimal criteria to determine the number of movements is a key limitation of use of this parameter for skill assessment.

Limited progress has been made to quantify hand or instrument movement beyond motion analysis using the parameters above. The dimensionless squared jerk (DSJ) was designed to be less dependent on time and to place more emphasis on movement. In physics, jerk is defined as a rate of change for acceleration. Therefore, it is a derivative of acceleration with respect to time and distance and as such is the second derivative of velocity or the third derivative of position. Hogan and Sternad noted that jerk could well-quantify the smoothness of motion related to hand coordination, with superior thoroughness; as such the sensitivity needed to be dimensionless, so that there would be no natural dependency of movement duration, extent, and spurious peaks [4].

The dimensionless squared jerk (DSJ) has been accepted as an objective parameter to quantify hand motion in different disciplines, such as parkinsonism, kinetics, and optometry. [32–35] To date, there is no study in the published literature quantifying hand motion using DSJ during simulated arthroscopic surgery.

In this study, we compare the ability of conventional parameters (procedural time, total path length, multiple measures of acceleration, and number of movements) to differentiate between novices and experts performing simulated shoulder arthroscopic tasks. To improve objective assessment of arthroscopic performance, we evaluated the construct validity of DSJ. Our hypothesis is that DSJ can differentiate between novices and experts performing simulated shoulder arthroscopic tasks and can be used as a parameter to train and assess surgeons.

## 2. Methods

### 2.1. Participants

All procedures performed in studies involving human participants were in accordance with the ethical standards of the institutional and/or national research committee and with the 1964 Helsinki Declaration and its later amendments or comparable ethical standards. Institutional Review Board was obtained from Asan Medical Center prior to study (no. 2017-0292). Informed consent was obtained from all individual participants included in the study. The two groups in this study were the novice group (no hands-on experience of arthroscopic surgery) and the expert group (shoulder arthroscopy consultant). A test study was performed to calculate the expected means of the performance parameters in both groups. A priori power analysis showed a minimum of 51 attempts in each group would be sufficiently powered (80%) at a significance level of 0.05. Twelve volunteers participated in this study. These included six residents (novice group) and six consultants (expert group). Each participant performed the simulated arthroscopic tasks ten times. All the participants were right handed; therefore, they controlled the arthroscope with the left hand and maneuvered the surgical instruments with their right hand.

### 2.2. Experiment Setup and Protocol

**Figure 1:**
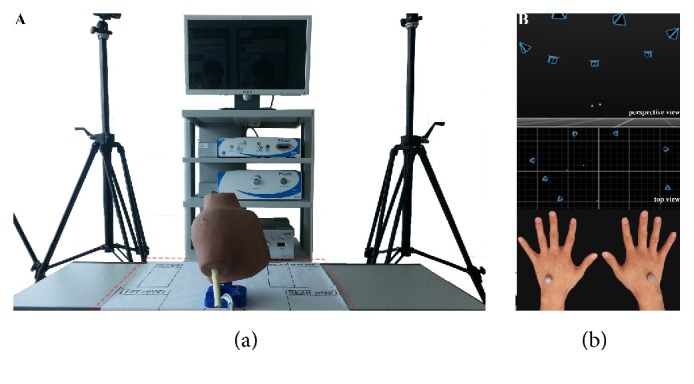
Experimental setup. (a) Standardized preparation tool (red dash lines). (b) Motion capture system configuration. Yellow dots are reflective markers. Blue outlined prisms are optical cameras.

Each participant performed three simulated arthroscopic tasks with a standard 30° angle arthroscope with 105° field of view (Conmed–Linvatec Corporation, Largo, FL). Both groups performed the experiment under the same experimental protocols and design and were evaluated with an optical tracking system. The phantom model, arthroscope, and surgical instruments were arranged in accordance with their predesigned places on the preparation table (Figure 1(a)).

The optical tracking system consisted of seven large-volume-motion-capture cameras (Prime 41; Natural Point, Inc., Corvallis, OR, USA). These were organized in a circular order to ensure their ability to capture two reflective markers. The markers were attached on the dorsal aspect of hands of the participants, at the mid-shaft point of the third metacarpal (Figure 1(b)). Marker trajectory was recorded with an associated tracking software (Motive: Tracker; Natural Point, Inc.) at a sampling rate of 120 Hz.

**Figure 2:**
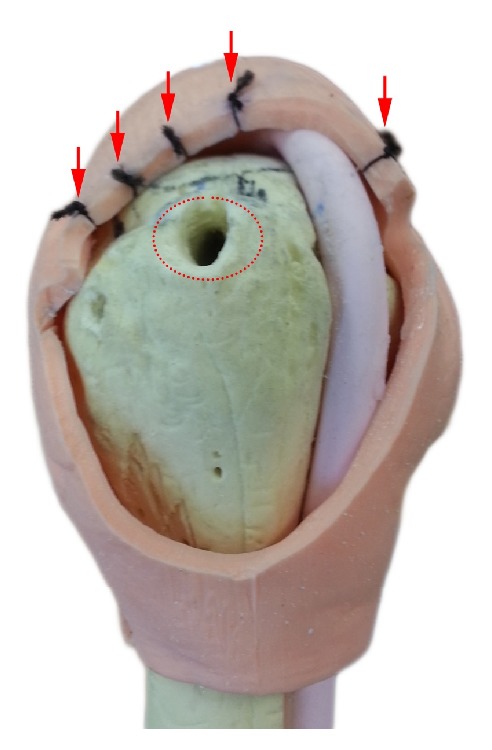
Modified human phantom shoulder model. Red-dots circle is a predesigned suture anchor site and red arrows are five predetermined points along the lateral border of rotator cuff.

A human shoulder phantom model (Arthrex Inc., Naples, FL, USA) was modified for shoulder arthroscopic simulation purposes. Five black silks were sutured at five different predetermined sites along the torn lateral border of the simulated rotator cuff (Figure 2).

All participants gave consent and were briefed about the experimental shoulder arthroscopic tasks. Each participant performed three validated shoulder arthroscopic tasks sequentially (Video 1) [2, 16]. Video is available as supplementary material. First, each participant touched five points along the rotator cuff with a grasper. Second, each participant inserted an anchor at a predetermined hole on the footprint of the rotator cuff on the greater tuberosity. Third, each participant pulled sutures through the anterior portal with grasper.

Participants placed their hand in a predetermined location on the preparation table before the start of the task and replaced to the initial position after completion of the task.

The optical tracking system was utilized to record three-dimensional (3D) movement of hand. Data were recorded from the time when the hands were off the preparation tool up to the time when all the surgical instruments were replaced to their initial locations for each task. All data generated or analyzed during this study are included within this article.

### 2.3. Data Collection and Analysis

The optical tracking system recorded the 3D position data (x, y, z) of each marker as a function of time. Total procedural time was calculated by adding the time for the three tasks cumulatively, without any intertask time. The total path length was defined as the distance traveled by the participant's hands during the three tasks (without any intertask distance). The acceleration parameter was analyzed in two ways: 1, computation of average acceleration; 2, computation of the maximum acceleration. The number of movements was defined by changes in velocity with respect to time, according to three threshold values: 1, acceleration above 10 m/s^2^; 2, acceleration above minimum acceleration of each participant; 3, acceleration above average acceleration of each participant.

Each participant's total DSJ, which was the jerk without dimensions, was calculated with the following formula [4, 36, 37]:

(1) $$\begin{array}{c} \left(\;\overset{t_{2}}{\underset{t_{1}}{\int }}x^{\prime \prime \prime }{\left(\;t\;\right)}^{2}dt\;\right)*\frac{D^{3}}{v_{mean}^{2}} \end{array}$$

where $\dddot{x}{\left(t\right)}^{2}dt$ is squared jerk, D is the movement's duration, and *v*_*mean*_ is the movement's average velocity which was calculated using the 3D position data. The formula for calculating the DSJ was chosen based on the previous study by Hogan and Stenard [4]. We confine our observation to the earlier measure which has been used in the previous studies. [36, 37]

A Wilcoxon-Mann-Whitney Test was performed to analyze the differences in each parameter. Level of significance was set at 0.05 (*p* value).

## 3. Results

There were highly significant differences (*p* < 0.001) between novices and experts when assessed using time, number of movements (minimum acceleration, average acceleration), and DSJ. A significant difference was observed in average acceleration (*p* = 0.050) and range of acceleration (*p* = 0.046). No significant difference was observed in number of movements (10 m/s^2^) (p = 0.371), maximum acceleration (*p *= 0.545), or total path length (*p *= 0.395) (Table 1). Master data table is available as supplementary material. The main result of this study has been presented at the 27th SECEC-ESSSE Congress, held in Berlin (Germany), September 13-16, 2017. [38]

**Figure 3:**
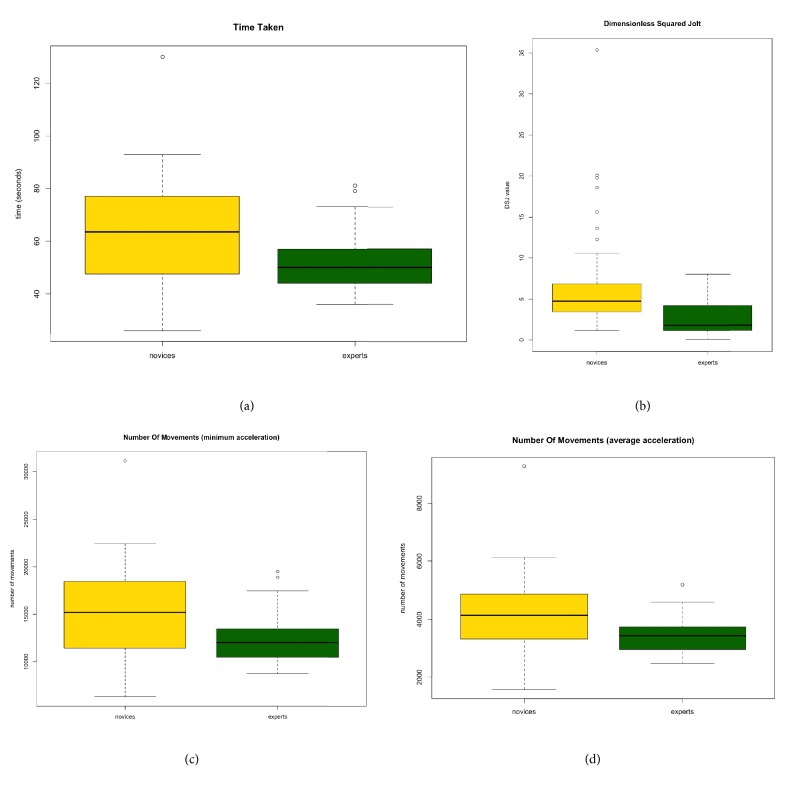
Whisker plots for (a) time, (b) dimensionless squared jerk, (c) number of movements (minimum acceleration), and (d) number of movements (average acceleration).

Consultants were significantly quicker to complete all tasks and had a faster average and range of acceleration. They also had a highly significant lower DSJ indicating that consultants had less unwanted and more purposeful movements than novices. Novices had significantly more number of movements when the threshold was defined using minimum and average acceleration. Using 10m/s^2^ as the acceleration threshold to define a movement [16], there was a tendency that novices had more movements, but this did not reach significance (Figures 3(a), 3(b), 3(c), and 3(d)).

## 4. Discussion

We have shown that DSJ as an objective parameter achieves construct validity in differentiating between novices and experts performing simulated shoulder arthroscopic tasks. This is logical as other studies have shown motion analysis to be a valid assessment tool in determining skill level. [12, 39–42]

Our results show that experts performed simulated arthroscopic tasks faster than novices in keeping with other studies [12, 43]. However, we would not draw the conclusion that the fastest surgeons are the best surgeons as procedural time alone does not give information on movement control or quantify risk of unnecessary iatrogenic injury. We did see a significantly larger range of acceleration in the experts compared to the novices whilst other studies reported the experts proceeded with a higher velocity [12, 28, 41, 42, 44].

Number of movements may help quantify risk of iatrogenic injury. In this study, we observed novices consistently had more hand movements. This is in keeping with other studies which also showed the novice usually will demonstrate unnecessary hand movements compared to experts [41, 44].

Using number of movements as an objective parameter to measure arthroscopic performance is difficult. This is partly because of spurious peaks that may occur because of two or more submovements [45, 46] and partly due to there being no consensus on the definition of a purposeful movement. We have evaluated three methods of defining a purposeful movement and support Jung et al. definition of a purposeful movement: one that results in an acceleration that exceeds the threshold set by the minimum acceleration of the participant [16]. This achieved statistical significance along with a purposeful movement being defined as an acceleration that exceeds average acceleration of the participant.

Defining a purposeful movement can be avoided by using DSJ as it is a parameter that is based on rate of acceleration and thus is independent of any threshold value. This allows DSJ to provide a measure of deliberate hand movements only by taking cognizance of the changes in acceleration (jerk) and the area under the jerk's curve to eliminate the potential bias induced by spurious peaks [4, 45, 46].

Another parameter that has been used to differentiate skill level of surgeons for arthroscopy is total path length [12, 43]. In this study, the expert group hand motion tracked over a longer total path length as compared to that in the novice group. We postulate that longer total path length may reflect the care taken to avoid iatrogenic damage to intra-articular structures in this anatomical simulated arthroscopic study. The shortest path between 2 points is a straight line, but to avoid collision, a longer nonlinear path may have been adopted by the experts. Further studies that incorporate collision data alongside motion analysis would help investigate this further.

### 4.1. Limitations

We accept that this study has several limitations. First, the number of participants in each group was low. Second, we only considered three shoulder arthroscopy tasks for this analysis, whilst there are numerous techniques and skills utilized during arthroscopic surgery. Future studies need to expand the number of standardized arthroscopic tasks, as we could not assess the novices' ability to perform complicated tasks without some training, thereby interfering with the results. Third, the hand motions were represented only by two markers. Such simplified motions cannot analyze the wrist, forearm, or elbow motion. Fourth, the assumption is that the simulated arthroscopic tasks correlate with intraoperative performance. Fifth, there is no clear definition of an expert arthroscopist. Lastly, we acknowledged that there were several methods proposed in quantifying smoothness of movement. Nevertheless, we felt that jerk-based measurement proposed by previous study by Hogan et al. would meet our study's purposes [4].

## 5. Conclusion

DSJ is an objective parameter that can differentiate experts and novices at simulated shoulder arthroscopy. Modern day training requires objective skills assessment to support competency-based curricula, and the DSJ can function as a useful objective performance parameter to measure deliberate movements alongside other motion analysis parameters.

## Figures and Tables

**Table 1:** The mean of the 10 performances stratified by participant and group (novice versus consultant). Wilcoxon-Mann-Whitney Test *p* values between groups. Min. acc.: minimum acceleration; Avg. acc.: average acceleration; Nov: novice. *∗∗* = statistically highly significant. *∗* = statistically significant.

	Time taken (*s*)		Average acceleration (*m/s*^*2*^)		Maximum acceleration (*m/s*^*2*^)		No. of movements (>10*m/s*^*2*^)		No. of movements (*min. acc.*)		No. of movements (*avg. acc.*)		Total path length (*m*)		Range of acceleration (*m/s*^*2*^)		DSJ	
	Nov	Expert	Nov	Expert	Nov	Expert	Nov	Expert	Nov	Expert	Nov	Expert	Nov	Expert	Nov	Expert	Nov	Expert
1	62.29	41.86	0.56	0.45	14.37	5.17	13.91	0.00	14463.16	10040.20	3657.00	3068.70	15.52	8.86	14.37	5.17	3.03	2.83
2	74.73	52.32	0.83	0.47	44.73	5.69	27.20	0.00	11336.00	12552.60	3008.10	3805.70	16.47	10.54	44.73	5.69	6.61	5.32
3	43.46	46.15	0.52	0.99	8.28	85.62	0.00	41.30	13545.20	11454.60	3899.10	3508.40	13.92	13.36	8.28	85.62	3.84	1.82
4	55.99	54.15	0.61	1.13	21.43	29.80	19.21	5.20	16964.56	12989.50	4780.30	3351.60	13.05	22.37	21.43	29.80	5.31	2.08
5	51.84	51.84	0.48	1.80	6.20	38.06	0.00	4.80	12844.00	12435.30	3865.80	3226.20	10.69	28.41	6.20	38.06	4.89	1.99
6	87.60	63.53	1.19	0.62	96.28	19.43	24.30	2.20	20873.30	14395.50	5667.30	3619.00	29.99	29.43	96.28	64.13	15.22	1.76
Mean	62.56	51.64	0.70	0.91	31.88	30.63	14.10	8.92	15004.37	12311.28	4146.27	3429.93	16.61	18.83	31.88	38.08	6.48	2.63
Standard deviation	19.40	10.73	0.53	0.58	104.69	37.82	32.13	17.77	4653.71	2574.73	1291.92	568.00	6.86	9.01	104.69	47.21	5.75	2.06
p value	<0.001*∗∗*		0.050∗		0.545		0.371		<0.001*∗∗*		<0.001*∗∗*		0.395		0.046*∗*		<0.001*∗∗*	

## Data Availability

The data used to support the findings of this study are available from the corresponding author upon request.

## Supplementary Materials

Supplementary Materials Video 1 in Methods and Master data table in Results are available as supplementary materials. Video shows three standardized arthroscopic tasks for each participant to complete. The performances data by participant and group (novice versus consultant). Wilcoxon-Mann-Whitney Test p values between groups.

## References

[1] Hodgins J. L., Veillette C. (2013). Arthroscopic proficiency: methods in evaluating competency. *BMC Medical Education*.

[2] Darzi A., Smith S., Taffinder N. (1999). Assessing operative skill. Needs to become more objective. *BMJ*.

[3] Milburn J. A., Khera G., Hornby S. T., Malone P. S., Fitzgerald J. E. (2012). Introduction, availability and role of simulation in surgical education and training: review of current evidence and recommendations from the Association of Surgeons in Training. *International Journal of Surgery*.

[4] Hogan N., Sternad D. (2009). Sensitivity of Smoothness Measures to Movement Duration, Amplitude, and Arrests. *Journal of Motor Behavior*.

[5] Mabrey J. D., Gillogly S. D., Kasser J. R. (2002). Virtual reality simulation of arthroscopy of the knee.. *Arthroscopy : the journal of arthroscopic & related surgery : official publication of the Arthroscopy Association of North America and the International Arthroscopy Association*.

[6] Alvand A., Logishetty K., Middleton R., Khan T., Jackson W. F., Price A. J. (2013). Validating a global rating scale to monitor individual resident learning curves during arthroscopic knee meniscal repair. *Arthroscopy: The Journal of Arthroscopic & Related Surgery*.

[7] Howells N. R., Gill H. S., Carr A. J., Price A. J., Rees J. L. (2008). Transferring simulated arthroscopic skills to the operating theatre. *The Journal of Bone & Joint Surgery*.

[8] Insel A., Carofino B., Leger R., Arciero R., Mazzocca A. D. (2009). The development of an objective model to assess arthroscopic performance. *The Journal of Bone & Joint Surgery—American Volume*.

[9] Koehler R., John T., Lawler J., Moorman C., Nicandri G. (2015). Arthroscopic training resources in orthopedic resident education. *The Journal of Knee Surgery*.

[10] Martin J. A., Regehr G., Reznick R. (1997). Objective structured assessment of technical skill (OSATS) for surgical residents. *British Journal of Surgery*.

[11] Middleton R. M., Baldwin M. J., Akhtar K., Alvand A., Rees J. L. (2016). Which global rating scale?: A comparison of the ASSET, BAKSSS, and IGARS for the assessment of simulated arthroscopic skills. *Journal of Bone and Joint Surgery - American Volume*.

[12] Howells N. R., Brinsden M. D., Gill R. S., Carr A. J., Rees J. L. (2008). Motion analysis: a validated method for showing skill levels in arthroscopy. *Arthroscopy: The Journal of Arthroscopic & Related Surgery*.

[13] Sidhu R. S., Grober E. D., Musselman L. J., Reznick R. K. (2004). Assessing competency in surgery: Where to begin?. *Surgery*.

[14] Alvand A., Auplish S., Gill H., Rees J. (2011). Innate arthroscopic skills in medical students and variation in learning curves. *The Journal of Bone & Joint Surgery*.

[15] Fatima H., Rex D. K., Rothstein R. (2008). Cecal Insertion and Withdrawal Times With Wide-Angle Versus Standard Colonoscopes: A Randomized Controlled Trial. *Clinical Gastroenterology and Hepatology*.

[16] Jung K., Kang D., Kekatpure A. L., Adikrishna A., Hong J., Jeon I. (2016). A new wide-angle arthroscopic system: a comparative study with a conventional 30° arthroscopic system. *Knee Surgery, Sports Traumatology, Arthroscopy*.

[17] Kim K., Kim D., Matsumiya K., Kobayashi E., Dohi T. Wide FOV Wedge Prism Endoscope.

[18] Kim S.-i., Suh T. S. World Congress on Medical Physics and Biomedical Engineering 2006.

[19] Pellisé M., Fernández–Esparrach G., Cárdenas A. (2008). Impact of Wide-Angle, High-Definition Endoscopy in the Diagnosis of Colorectal Neoplasia: A Randomized Controlled Trial. *Gastroenterology*.

[20] Rex D. K., Chadalawada V., Helper D. J. (2003). Wide angle colonoscopy with a prototype instrument: Impact on miss rates and efficiency as determined by back-to-back colonoscopies. *American Journal of Gastroenterology*.

[21] Aggarwal R., Grantcharov T., Moorthy K., Milland T., Darzi A. (2008). Toward feasible, valid, and reliable video-based assessments of technical surgical skills in the operating room. *Annals of Surgery*.

[22] Doyle J. D., Webber E. M., Sidhu R. S. (2007). A universal global rating scale for the evaluation of technical skills in the operating room. *The American Journal of Surgery*.

[23] Eubanks T. R., Clements R. H., Pohl D. (1999). An objective scoring system for laparoscopic cholecystectomy. *Journal of the American College of Surgeons*.

[24] Vassiliou M. C., Feldman L. S., Andrew C. G. (2005). A global assessment tool for evaluation of intraoperative laparoscopic skills. *The American Journal of Surgery*.

[25] Vassiliou M. C., Kaneva P. A., Poulose B. K. (2010). Global assessment of gastrointestinal endoscopic skills (GAGES): A valid measurement tool for technical skills in flexible endoscopy. *Surgical Endoscopy*.

[26] Howells N. R., Auplish S., Hand G. C., Gill H. S., Carr A. J., Rees J. L. (2009). Retention of arthroscopic shoulder skills learned with use of a simulator: Demonstration of a learning curve and loss of performance level after a time delay. *The Journal of Bone & Joint Surgery*.

[27] Jackson W. M., Khan T., Alvand A. (2012). Learning and Retaining Simulated Arthroscopic Meniscal Repair Skills. *The Journal of Bone and Joint Surgery-American Volume*.

[28] Gomoll A. H., O'Toole R. V., Czarnecki J., Warner J. J. P. (2007). Surgical experience correlates with performance on a virtual reality simulator for shoulder arthroscopy. *The American Journal of Sports Medicine*.

[29] Martin K. D., Belmont P. J., Schoenfeld A. J., Todd M., Cameron K. L., Owens B. D. (2011). Arthroscopic Basic Task Performance in Shoulder Simulator Model Correlates with Similar Task Performance in Cadavers. *The Journal of Bone and Joint Surgery-American Volume*.

[30] Pedowitz R. A., Esch J., Snyder S. (2002). Evaluation of a virtual reality simulator for arthroscopy skills development. *Arthroscopy: The Journal of Arthroscopic & Related Surgery*.

[31] Smith S., Wan A., Taffmder N., Read S., Emery R., Darzi A. (1999). Early experience and validation work with procedicus va - The prosolvia virtual reality shoulder arthroscopy trainer. *Studies in Health Technology and Informatics*.

[32] Flash T., Hogan N. (1985). The coordination of arm movements: an experimentally confirmed mathematical model. *The Journal of Neuroscience*.

[33] Ketcham C. J., Seidler R. D., Van Gemmert A. W., Stelmach G. E. (2002). Age-related kinematic differences as influenced by task difficulty, target size, and movement amplitude. *The Journals of Gerontology Series B: Psychological Sciences and Social Sciences*.

[34] Teulings H. L., Contreras-Vidal J. L., Stelmach G. E., Adler C. H. (1997). Parkinsonism reduces coordination of fingers, wrist, and arm in fine motor control. *Experimental Neurology*.

[35] Wyatt H. J. (1998). Detecting saccades with jerk. *Vision Research*.

[36] Takada K., Yashiro K., Takagi M. (2006). Reliability and sensitivity of jerk-cost measurement for evaluating irregularity of chewing jaw movements. *Physiological Measurement*.

[37] Yashiro K., Nakamura T., Mizumori T., Yatani H., Takada K. Clinical validity of measuring jerk-cost of jaw movement during speech: Effect of mouthguard design on smoothness of jaw movements.

[38] Kholinne E. A. A., Gandhi M. J., Hong H. P., Jeon I. H. The Dimensionless Squarred Jolt (DSJ) - A novel objective parameter that improves assessment of hand motion analysis during shoulder arthroscopy.

[39] Datta V., Chang A., Mackay S., Darzi A. (2002). The relationship between motion analysis and surgical technical assessments. *The American Journal of Surgery*.

[40] Mason J. D., Ansell J., Warren N., Torkington J. (2013). Is motion analysis a valid tool for assessing laparoscopic skill?. *Surgical Endoscopy*.

[41] Nasr A., Carrillo B., Gerstle J. T., Azzie G. (2014). Motion analysis in the pediatric laparoscopic surgery (PLS) simulator: Validation and potential use in teaching and assessing surgical skills. *Journal of Pediatric Surgery*.

[42] Tashiro Y., Miura H., Nakanishi Y., Okazaki K., Iwamoto Y. (2009). Evaluation of Skills in Arthroscopic Training Based on Trajectory and Force Data. *Clinical Orthopaedics and Related Research*.

[43] Kirby G. S. J., Guyver P., Strickland L. (2014). Assessing arthroscopic skills using wireless elbow-worn motion sensors. *Journal of Bone and Joint Surgery - American Volume*.

[44] Datta V., Mackay S., Mandalia M., Darzi A. (2001). The use of electromagnetic motion tracking analysis to objectively measure open surgical skill in the laboratory-based model. *Journal of the American College of Surgeons*.

[45] Drakesmith M., Caeyenberghs K., Dutt A., Lewis G., David A. S., Jones D. K. (2015). Overcoming the effects of false positives and threshold bias in graph theoretical analyses of neuroimaging data. *NeuroImage*.

[46] Rohrer B., Hogan N. (2006). Avoiding spurious submovement decompositions II: A scattershot algorithm. *Biological Cybernetics*.

